# An Adaptor Molecule Afadin Regulates Lymphangiogenesis by Modulating RhoA Activity in the Developing Mouse Embryo

**DOI:** 10.1371/journal.pone.0068134

**Published:** 2013-06-26

**Authors:** Takashi Majima, Keisuke Takeuchi, Keigo Sano, Masanori Hirashima, Dimitar P. Zankov, Miki Tanaka-Okamoto, Hiroyoshi Ishizaki, Jun Miyoshi, Hisakazu Ogita

**Affiliations:** 1 Department of Molecular Biology, Osaka Medical Center for Cancer and Cardiovascular Disease, Osaka, Japan; 2 Division of Molecular Medical Biochemistry, Department of Biochemistry and Molecular Biology, Shiga University of Medical Science, Shiga, Japan; 3 Division of Vascular Biology, Department of Physiology and Cell Biology, Kobe University Graduate School of Medicine, Hyogo, Japan; Osaka University Graduate School of Medicine, Japan

## Abstract

Afadin is an intracellular binding partner of nectins, cell-cell adhesion molecules, and plays important roles in the formation of cell-cell junctions. Afadin-knockout mice show early embryonic lethality, therefore little is known about the function of afadin during organ development. In this study, we generated mice lacking afadin expression in endothelial cells, and found that the majority of these mice were embryonically lethal as a result of severe subcutaneous edema. Defects in the lymphatic vessels of the skin were observed, although the morphology in the blood vessels was almost normal. Severe disruption of VE-cadherin-mediated cell-cell junctions occurred only in lymphatic endothelial cells, but not in blood endothelial cells. Knockout of afadin did not affect the differentiation and proliferation of lymphatic endothelial cells. Using *in vitro* assays with blood and lymphatic microvascular endothelial cells (BMVECs and LMVECs, respectively), knockdown of afadin caused elongated cell shapes and disruption of cell-cell junctions among LMVECs, but not BMVECs. In afadin-knockdown LMVECs, enhanced F-actin bundles at the cell periphery and reduced VE-cadherin immunostaining were found, and activation of RhoA was strongly increased compared with that in afadin-knockdown BMVECs. Conversely, inhibition of RhoA activation in afadin-knockdown LMVECs restored the cell morphology. These results indicate that afadin has different effects on blood and lymphatic endothelial cells by controlling the levels of RhoA activation, which may critically regulate the lymphangiogenesis of mouse embryos.

## Introduction

The formation of intercellular junctions is a fundamental cellular function that is crucial for tissue morphogenesis, including angiogenesis and vasculogenesis, in various animals. Several kinds of junctional apparatuses, such as adherens junctions (AJs), exist at cell-cell adhesion sites [1]. AJs are present in epithelial cells, endothelial cells and fibroblasts, and act as mechanically adhesive machinery between opposing cells. AJs consist of multiple cell adhesion molecules (CAMs) and intracellular scaffolding molecules that directly or indirectly link CAMs to the actin cytoskeleton, resulting in the formation of complex structures that make firm adhesive connections between cells. Cadherins are the major CAMs at AJs, and their adhesion activity is Ca^2+^-dependent [2]. The cadherin super family is classified into several groups including classical cadherins, desmosomal cadherins, and protocadherins. Classical cadherins include E-cadherin and VE-cadherin, which are expressed in epithelial cells and vascular endothelial cells, respectively, and only mediate homophilic *trans*-interactions with each other [3]. β-Catenin interacts with the cytoplasmic region of cadherins and directly binds to α-catenin, which is tethered to actin filaments (F-actin). Thus, cadherins are anchored to the actin cytoskeleton via these catenins. A nectin family consisting of four members, nectin-1 through nectin-4, are another kind of CAMs, which localize at AJs of various cell-cell adhesion sites including those of endothelial cells [4]. Nectins mediate both homophilic and heterophilic *trans*- interactions with each other among the family members. Similar to cadherins, nectins are connected to the actin cytoskeleton by an intracellular binding partner, afadin [5]. The nectin-afadin and cadherin-catenin complexes play important roles, both cooperatively and independently, in the formation of cell-cell junctions including AJs [6], [7].

Our investigations revealed that afadin has multiple functions in cells. Other than its important role in the formation of AJs together with nectins, afadin regulates cell polarization and tight junction (TJ) formation by associating with a TJ component, ZO-1, in epithelial cells [8]. TJs are located on the lateral membranes of epithelial and endothelial cells, and limit the paracellular passage of soluble molecules across cellular sheets [9], [10]. In addition, afadin promotes cell survival and regulates directional cell movement of individually moving cells in a nectin-independent manner [11]–[13]. These functions of afadin have been confirmed in cultured cells and embryoid bodies generated from embryonic stem cells [14].

Afadin-knockout mice die *in utero* [15], [16], and afadin-deficient embryos show developmental defects at stages during and after gastrulation. In these embryos, gastrulation itself appears to occur because the mesoderm can be generated. However, the migration of mesodermal cells is suppressed and the ectoderm is disorganized by improperly formed cell-cell junctions, resulting in loss of somites and other structures derived from both the ectoderm and mesoderm, and embryonic death at around embryonic day (E) 8.5. Because of this early embryonic death, little is known about the role of afadin during organ development. Although some studies showed the importance of afadin for the endothelial barrier function and postnatal angiogenesis [17], [18], the role of afadin in the formation of vascular network during the developmental period remains to be elucidated. To address this issue, we generated afadin conditional knockout (cKO) mice in which the *afadin* gene was deleted specifically in endothelial cells by the Cre/loxP system, and then analyzed the mice, followed by *in vitro* experiments using cultured endothelial cells to reveal the molecular mechanisms of the phenomena observed in afadin cKO mice.

## Materials and Methods

### Generation of afadin cKO mice

Afadin-floxed mice (afadin^flox/flox^), in which exon 2 of the *afadin* gene was flanked by loxP sites, were generated as described previously and then backcrossed at least six times onto the C57BL/6 strain [19]. Tie2-Cre transgenic mice (C57BL/6 background) and ROSA26R mice were purchased from The Jackson Laboratory. To obtain endothelial cell-specific afadin cKO mice, in the first cross, Tie2-Cre transgenic mice were mated with afadin^flox/flox^ mice, and then 50% of the offspring with the afadin^flox/+^;Tie2-Cre genotype were mated with afadin^flox/flox^ mice. Mice used in this study were housed 5 or less per cage in static microisolation caging in a specific pathogen-free facility of the Research Center for Animal Life Sciences at Shiga University of Medical Science and the Animal Center at Osaka Medical Center for Cancer and Cardiovascular Diseases with being careful for animal welfare. Mice were able to freely access to standard chows and sterilized water. The pregnant female mice and mice at P21 were euthanized by cervical dislocation, and mice at P0 were euthanized by CO_2_ inhalation. The animal experimental procedures conducted in this study were reviewed and approved by the Shiga University of Medical Science Animal Care and Use Committee, and the Review Committee of the Osaka Medical Center for Cancer and Cardiovascular Diseases.

### Genotyping

Genotyping was performed by PCR using DNA isolated from the yolk sacs of embryos or from tail biopsies of postnatal mice. To identify the floxed afadin allele, forward and reverse primers (5′-TAGGAGGAACATGTGAAAGG-3′ and 5′-CCATGAAACTCCAA ATCCTA-3′, respectively) were used for PCR to amplify 390 bp (homozygous) or 390/293 bp (heterozygous) fragments. The Tie2-Cre transgene was detected by PCR using the following primers: 5′-GAACGTGCAAAACAGGCTCTA-3′ (forward) and 5′-AGTTACCCCCAGGCTAAGTGC-3′ (reverse) to generate a 270 bp product.

### Antibodies

The antibodies (Abs) listed below were purchased from commercial sources: a rabbit anti-afadin polyclonal Ab (pAb) (Sigma-Aldrich, St. Louis, MO, USA), rat anti-LYVE-1 pAb (RELIATech, Wolfenbüttel, Germany), Armenian hamster anti-PECAM-1 monoclonal (mAb) (clone 2H8; Endogen, Woburn, MA, USA), rat anti-PECAM-1 mAb (clone Mec13.3; BD Pharmingen, San Jose, CA, USA), rabbit anti-Prox1 pAb (Covance, Princeton, NJ, USA), Syrian hamster anti-podoplanin mAb (clone 8.1.1; Developmental Studies Hybridoma Bank, Iowa City, IA, USA), rat anti-VE-cadherin mAb (clone 11D4.1; BD Pharmingen), rabbit anti-connexin 40 pAb (Alpha diagnostic international, San Antonio, TX, USA), goat anti-EphB4 pAb (R&D Systems, Minneapolis, MN, USA), rabbit anti-glyceraldehyde-3-phosphate dehydrogenase (GAPDH) mAb (clone 14C10; Cell Signaling Technology, Danvers, MA, USA), rabbit anti-cleaved caspase-3 pAb (Cell signaling Technology), mouse anti-VE-cadherin mAb (clone 75; BD Pharmingen) and rabbit anti-RhoA pAb (Santa Cruz Biotechnology, Santa Cruz, CA, USA). For immunofluorescence microscopy, we used Alexa Fluor 488- or Cy3-conjugated secondary Abs (Invitrogen, Carlsbad, CA, USA, or Jackson ImmunoResearch, West Grove, PA, USA), rhodamine-phalloidin (Invitrogen) and Hoechst 33258 (Invitrogen).

### Immunofluorescence microscopy

Immunohistochemical analysis of the back skin obtained from embryos was performed as described previously [20]. Samples were observed under an Olympus FV-1000 (Olympus, Tokyo, Japan) confocal microscope. For immunofluorescence experiments using cultured endothelial cells, cells were fixed with 2% paraformaldehyde for 30 min, followed by 0.2% Triton X-100 permeabilization for 10 min, and then incubation with 1% bovine serum albumin in phosphate-buffered saline (PBS) for blocking. F-actin and nuclei were labeled with rhodamine-phalloidin and Hoechst 33258, respectively. Samples were observed under a Nikon C1si (Nikon, Tokyo, Japan) confocal microscope.

### Whole-mount staining

For whole mount LacZ staining, embryos obtained by crossing homozygous ROSA26R mice with Tie2-Cre mice were dissected and rinsed in PBS. After the yolk sac was removed, embryos were fixed in 2% paraformaldehyde with 0.05% glutaraldehyde at room temperature for 1 h, washed three times in LacZ wash buffer (PBS with 0.02% Nonidet P-40, and 2 mM MgCl_2_) at room temperature for 20 min, and then stained with an X-Gal staining solution (0.1% 5-bromo-4-chloro-3-indolyl-β-D-galactopyranoside, 5 mM K_3_Fe(CN)_6_, 5 mM K_4_Fe(CN)_6_, 2 mM MgCl_2_, and 0.02% Nonidet P-40) at 37°C overnight. Stained embryos were washed three times in LacZ wash buffer at room temperature for 20 min, and then postfixed in a 3.7% formalin solution at 4°C overnight. For whole-mount PECAM-1 staining of embryos, embryos were fixed in methanol/dimethyl sulfoxide (4∶1) at 4°C overnight. Endogenous peroxidase activity was quenched with 0.3% hydrogen peroxide in methanol at room temperature for 1 h, followed by rehydration in PBS containing 0.2% Triton X-100 (PBT) at 4°C for 30 min. PECAM-1 staining was performed as previously described [20]. Immunoreactivity was visualized with 0.025% 3,3-diaminobenzidine and 0.08% NiCl_2_ in PBT.

### BrdU incorporation assay

The BrdU incorporation assay was performed using a BrdU Cell Proliferation Assay Kit (Calbiochem, Darmstadt, Germany) and an Alexa Fluor 546-conjugated mouse anti-BrdU mAb (clone PRB-1; Invitrogen) according to the manufacturer's instructions. Briefly, pregnant mice were injected with BrdU (100 µg/g body weight) on E14.5. Fetuses were harvested at 2 h post-injection and the back skins of embryos were fixed with 4% paraformaldehyde, followed by BrdU/Prox1 double immunostaining.

### Cell culture and transfection

Normal human dermal blood and lymphatic microvascular endothelial cells (BMVECs and LMVECs, respectively) were purchased from Lonza (Walkersville, MD, USA) and cultured in EGM-2-MV BulletKit™ medium (Lonza). To knockdown the expression of afadin in these cells, a Stealth™ RNAi duplex (siRNA) against human afadin and a Stealth RNAi negative control duplex (Scramble) were purchased from Invitrogen and transfected into the cells with LipofectAMINE 2000 reagent (Invitrogen) according to the manufacturer's instructions. The sequence of siRNA against human afadin was as follows: 5′-CCCAAGACAUAAACCUGGAAUUGUU-3′. Mammalian expression vectors for enhanced green fluorescent protein (EGFP; pEGFP-C1, Clontech, Mountain View, CA, USA) or EGFP-tagged dominant-negative RhoA (pEGFP-RhoA-N19), in which a residue at amino acid 19 was replaced by asparagine (N), were transfected into cells together with RNAi duplexes.

### Pull-down assay to assess RhoA activation

The pull-down assay was carried out as described previously [21]. Briefly, the cells were lysed in Buffer A (50 mM Tris/HCl, pH 7.4, 150 mM NaCl, 5 mM MgCl_2_, 1% Nonidet P-40, 0.5% sodium deoxycholate, 0.1% SDS, 1 mM Na_3_VO_4_, 10 µg/ml leupeptin, 2 µg/ml aprotinin, and 10 µM *p*-amidinophenylmethylsulfonylfluoride) containing 30 µg Glutathione *S*-transferase-rhotekin, and then incubated at 2°C for 30 min. The cell extract was obtained by centrifugation at 20,000× *g* at 2°C for 10 min, and then incubated with 50 µl of 50% slurry Glutathione-Sepharose beads (GE Healthcare, Piscataway, NJ, USA) at 2°C for 1 h. After the beads were washed with Buffer A, proteins bound to the beads were eluted with SDS sample buffer and subjected to SDS-polyacrylamide gel electrophoresis, followed by western blotting with an anti-RhoA pAb.

## Results

### Ablation of afadin in Tie2-Cre-positive endothelial cells

**Figure 1:**
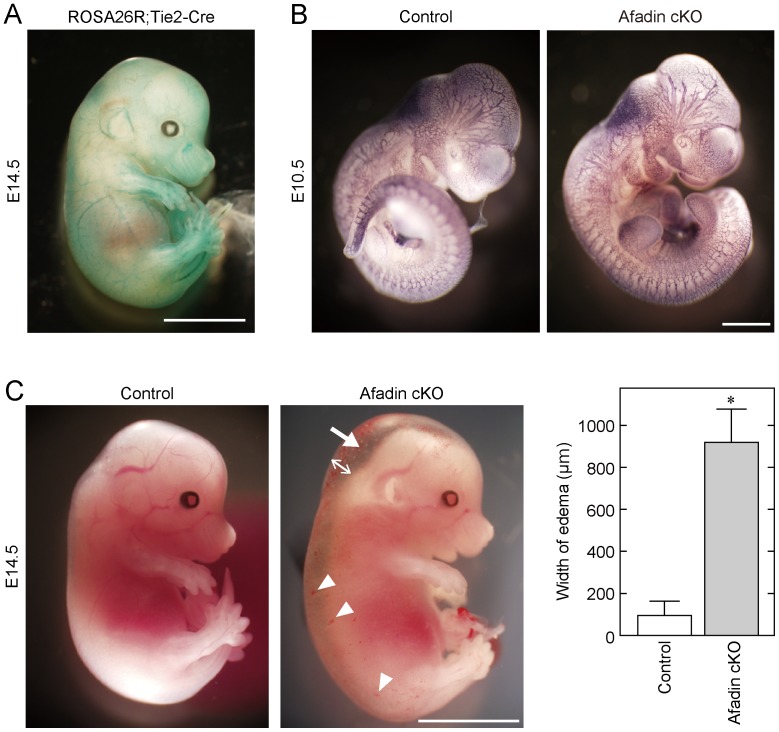
Severe subcutaneous edema in afadin cKO mice during embryogenesis. (**A**) Expression pattern of Cre recombinase. Embryos harboring the ROSA26R^+/−^;Tie2-Cre genotype at E14.5 were used for whole-mount LacZ staining. (**B**) No significant differences between afadin cKO mice and control mice in their body sizes and vascular structures at E10.5. Embryos at E10.5 were fixed and immunostained with an anti-PECAM-1 mAb. (**C**) Severe subcutaneous edema and superficial scattered hemorrhage were observed in afadin cKO mice at E14.5. An arrow and arrowheads indicate edema and hemorrhage, respectively. Bar graphs show the width of edema measured at the back of the neck (two-direction arrow), and error bars indicate the mean ±S.D. *, p<0.01 vs. Control. Scale bars in (**A**), (**B**) and (**C**) represent 5 mm, 1 mm and 5 mm, respectively.

We first confirmed the function of Cre recombinase in Tie2-Cre mice we used by analyzing the embryos obtained from crosses between Tie2-Cre mice and ROSA26R reporter mice (**Fig. 1A**). In the ROSA26R;Tie2-Cre embryo at E14.5, LacZ expression was detected specifically in vessels in a Cre-dependent manner, indicating the proper function of Cre recombinase in Tie2-Cre mice.

At P0, 186 viable pups were generated from crosses between afadin^flox/flox^ and afadin^flox/+^;Tie2-Cre mice. We obtained 69 afadin^flox/+^;Tie2-Cre pups, 53 afadin^flox/flox^ pups, and 55 afadin^flox/+^ pups, but only 9 afadin^flox/flox^;Tie2-Cre (afadin cKO) pups (**Table 1**). The numbers of afadin cKO pups born (P0) were much lower than those of pups with the other genotypes. A similar situation was observed with adult afadin cKO mice (P21). These results indicate that the majority of afadin cKO mice are embryonically lethal. Next, we attempted to identify the time of death of afadin cKO embryos by analyzing E9.5 to E16.5 mice. Until E13.5, afadin cKO embryos were indistinguishable from their control littermates in terms of gross morphology and vascular development (**Fig. 1B** **and data not shown**). The percentage of afadin cKO embryos at E14.5 and E15.5 was near the expected Mendelian ratio, but almost all embryos exhibited severe subcutaneous edema accompanied by superficial scattered hemorrhages (**Fig. 1C**), which was considered to result in embryonic lethality. Taken together, these results suggest that afadin regulates vascular development during the late stages of embryogenesis.

**Table 1:** Genotype and number of viable progeny from crosses between afadin^flox/+^;Tie2-Cre and afadin^flox/flox^ mice.

	Afadin^flox/flox^;Tie2-Cre			Afadin^flox/+^;Tie2-Cre		Afadin^flox/flox^		Afadin^flox/+^	
E9.5	15	(34.1)		15	(34.1)	11	(25.0)	3	(6.8)
E10.5	11	(25.0)		12	(27.3)	9	(20.5)	12	(27.3)
E11.5	3	(33.3)		3	(33.3)	2	(22.2)	1	(11.1)
E12.5	5	(20.8)		9	(37.5)	6	(25.0)	4	(16.7)
E13.5	2	(15.4)		6	(46.2)	2	(15.4)	3	(23.1)
E14.5	7	(10.8)	[6]	20	(30.8)	19	(29.2)	19	(29.2)
E15.5	10	(18.5)	[10]	18	(33.3)	16	(29.6)	10	(18.5)
E16.5	1	(3.6)		9	(32.1)	13	(46.4)	5	(17.9)
P0	9	(4.8)		69	(37.1)	53	(28.5)	55	(29.6)
P21	6	(3.6)		62	(36.7)	47	(27.8)	54	(32.0)

The percentage of each genotype relative to the total progeny at each embryonic and post natal day is shown in parentheses (). The number of mice with an abnormal phenotype is shown in brackets [ ].

### Abnormal lymphatic vessel formation in afadin cKO embryos

**Figure 2:**
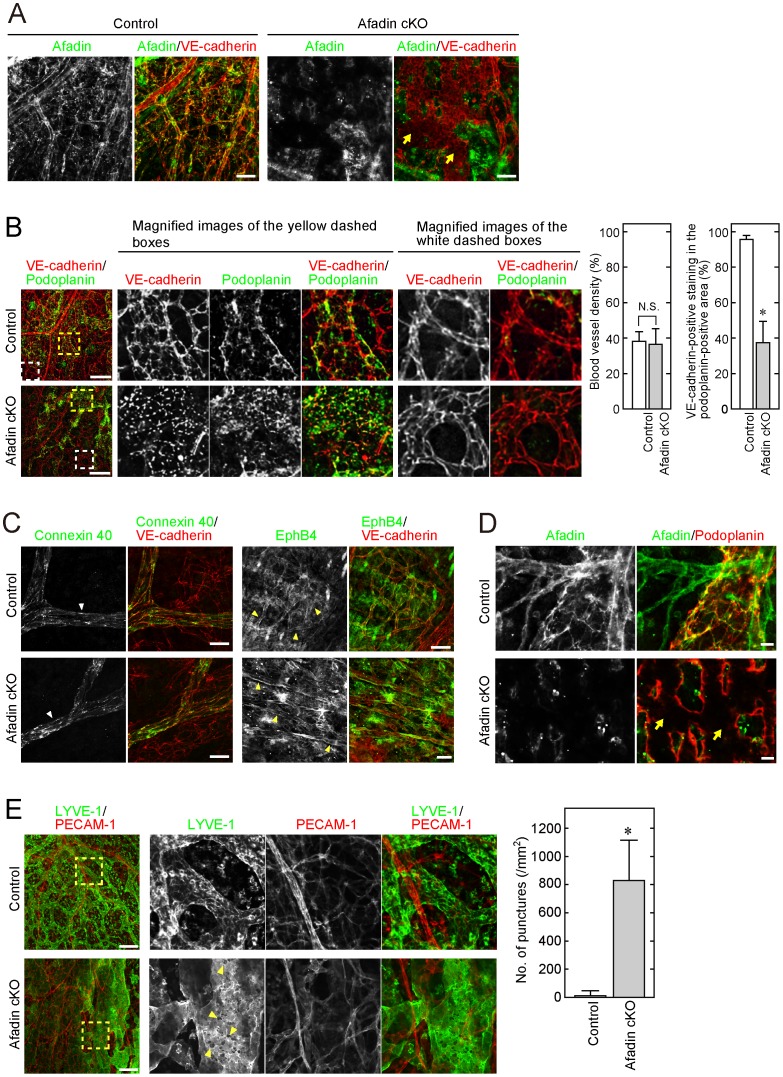
Disruption of cell-cell junctions on lymphatic, but not blood, vessel walls in afadin cKO mice. Back skins from control and afadin cKO embryos at E14.5 were fixed and immunostained with the indicated combination of Abs. Magnified images of the dashed boxes in (**B**) and (**E**) are also depicted. Arrows in (**A**) and (**D**) indicate the afadin-negative and VE-cadherin-positive area with patchy staining of VE-cadherin (**A**) and the afadin-negative and podoplanin-positive area with abnormal staining of podoplanin (**D**). White and yellow arrowheads in (**C**) indicate the arterial and venous vasculatures, respectively. Arrowheads in (**E**) indicate numerous punctures on lymphatic vessel walls. Bar graphs in (**B**) and (**E**) show the blood vessel density determined by VE-cadherin-positive and podoplanin-negative staining in the randomly selected areas (**B, left**), the percentage of VE-cadherin-positive staining in the podoplanin-positive areas (**B, right**) and the number of punctures in the LYVE-1-positive areas (**E**). Error bars indicate the mean ±S.D. at least three different samples. *, p<0.01 vs. Control. N.S., not statistically significant. Scale bars in (**A**), (**B**), (**C**), (**D**) and (**E**) represent 100 µm, 200 µm, 20 µm, 20 µm and 200 µm, respectively.

Before we further examined the abnormalities in afadin cKO mice during late embryogenesis, we verified loss of expression of afadin in the vasculature of afadin cKO mice. We immunostained the skin of embryos with an anti-afadin pAb and an anti-VE-cadherin mAb. VE-cadherin is a major CAM in endothelial cells and thus is a marker for endothelial cells [22]. Immunostaining of afadin was observed in both VE-cadherin-positive and -negative areas in control skin, whereas it was observed only in VE-cadherin-negative areas in afadin cKO skin (**Fig. 2A**). This indicates that the expression of afadin is deleted specifically in the endothelial cells of afadin cKO mice.

During the observation of this immunostaining, we noted that VE-cadherin staining was patchy in several areas of afadin cKO skin, compared with that in control skin (**Fig. 2A** **, arrows**). To determine what kind of endothelial cells showed such abnormal VE-cadherin staining in afadin cKO mice, we double immunostained the skin of embryos with an anti-VE-cadherin mAb and an anti-podoplanin pAb. Podoplanin is a membrane glycoprotein and a marker for lymphatic vessels [23]. VE-cadherin-positive and podoplanin-negative immunostaining, which indicates blood vessels, was not significantly different between afadin cKO and control embryos (**Fig. 2B** **, white dashed boxes**). The blood vessel density was not significantly different between the two types of embryos (**Fig. 2B** **, left bar graphs**). However, in lymphatic vessels identified by podoplanin-positive staining, VE-cadherin staining was quite different in afadin cKO embryos, compared with that in controls (**Fig. 2B** **, yellow dashed boxes and right bar graphs**). The speckled staining, instead of normal striated staining, of VE-cadherin was observed specifically in that area of afadin cKO embryos. No significant defects by endothelial cell-specific loss of afadin on blood vessels was further confirmed by double immunostaining with VE-cadherin and either an anti-connexin 40 antibody, an artery marker [24], or an anti-Eph B4 antibody, a vein marker [25]. The staining was similar between control and afadin cKO embryos (**Fig. 2C**). The expression of afadin in lymphatic vessels and loss of it in the vessels of afadin cKO mice were checked by immunostaining of afadin with podoplanin (**Fig. 2D**). These results indicate that lymphatic endothelial cells in afadin cKO mice have an abnormal phenotype due to loss of afadin. Because VE-cadherin plays an important role in the formation and maintenance of endothelial intercellular junctions, the cell-cell junctions in lymphatic vessels were considered to be disrupted during late embryogenesis of afadin cKO mouse embryos, leading to the occurrence of severe edema and embryonic lethality.

We further confirmed this abnormality with another combination of antibodies: an anti-PECAM-1 mAb and anti-LYVE-1 pAb. PECAM-1 is a marker for endothelial cells, and LYVE-1 is a marker for lymphatic vessels [23]. Similar to the above results, PECAM-1-positive and LYVE-1-negative blood vessels did not appear to be affected in both types of mice, whereas LYVE-1-positive lymphatic vessels were abnormally dilated in afadin cKO mice and showed numerous punctures on the vessel walls, which were likely caused by the disorganization of cell adhesion between lymphatic endothelial cells by loss of afadin (**Fig. 2E**).

### No differentiation abnormality or proliferation delay in afadin cKO embryos

**Figure 3:**
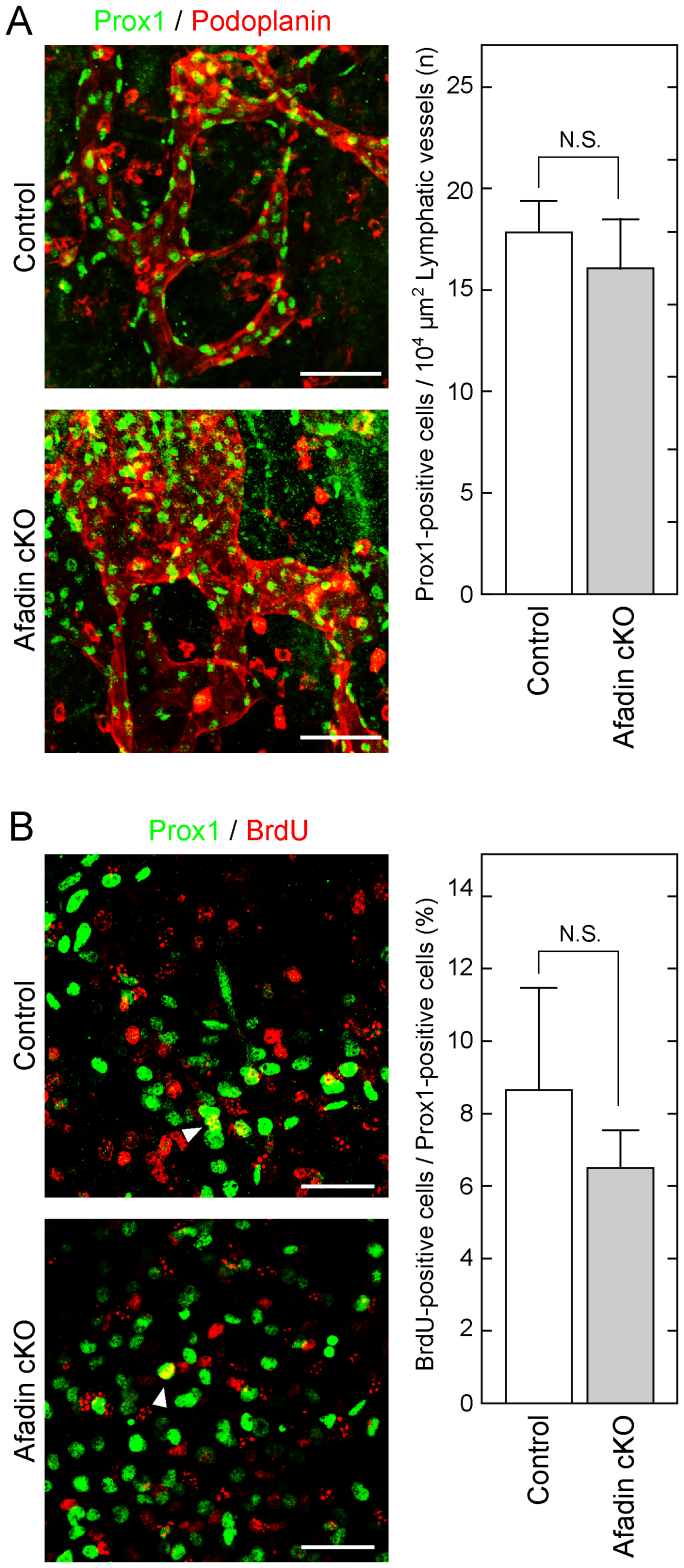
No significant difference in the differentiation and proliferation states between control and afadin cKO mice. Back skins from embryos at E14.5 were fixed and immunostained with the indicated Abs. Arrowheads in (**B**) indicate the cells double positive for Prox1 and BrdU. Scale bars in (**A**) and (**B**) represent 100 µm and 50 µm, respectively. Bar graphs show the number of Prox1-positive cells in the specific area of lymphatic vessels determined by podoplanin staining (**A**) and the percentage of BrdU-positive cells among Prox1-positive cells (**B**). Error bars indicate the mean ±S.D. from at least three independent experiments. N.S., not statistically significant.

To exclude the possibility that abnormal differentiation and/or proliferation caused the deteriorative phenotype in afadin cKO mice, immunostaining of Prox1, a lymphatic differentiation marker [26], and a BrdU incorporation assay were performed. The number of Prox1-positive cells in podoplanin-positive lymphatic vessels was similar between afadin cKO and control mice (**Fig. 3A**). The ratio of BrdU incorporation in Prox1-positive cells was also similar between these types of mice (**Fig. 3B**). These results suggest that afadin does not regulate differentiation or proliferation of endothelial cells during embryonic development.

### Mechanism of lymphatic endothelial cell-specific disruption of cell-cell junctions in afadin cKO embryos

**Figure 4:**
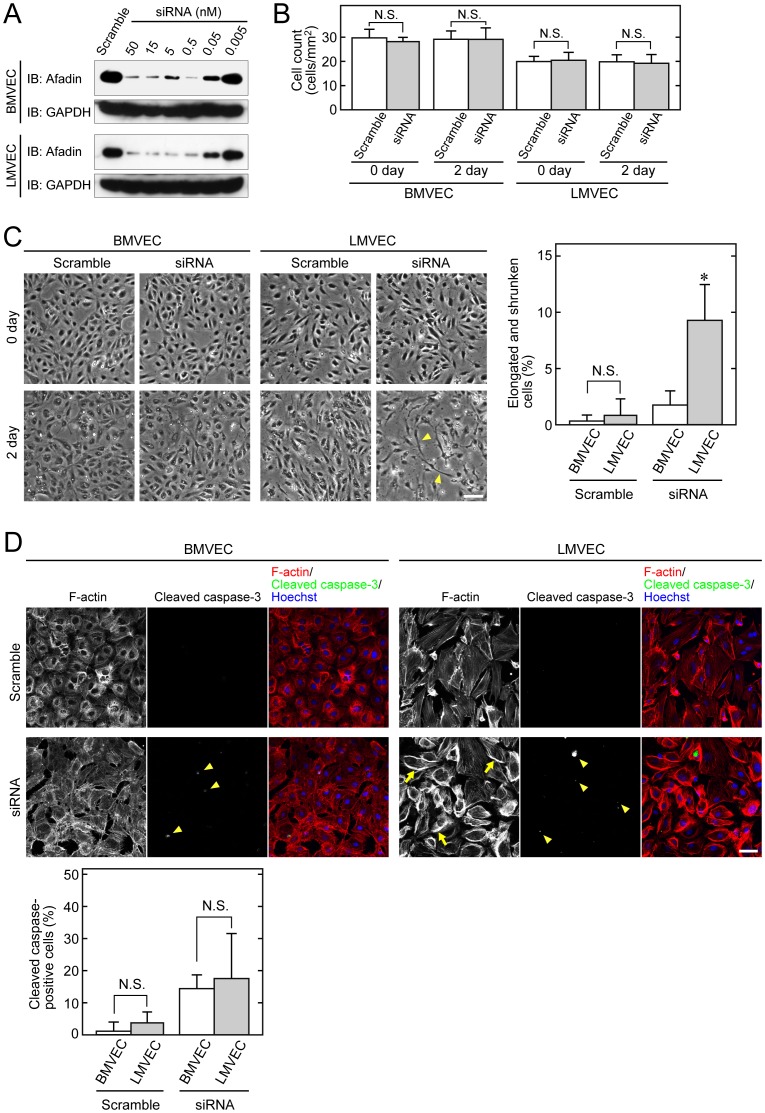
Lymphatic endothelial cell-specific morphological change by inactivation of afadin. (**A**) Knockdown of afadin in endothelial cells. BMVECs and LMVECs were transfected with the indicated concentration of siRNA against afadin or 50 nM control oligo-RNA (Scramble). Knockdown of afadin was evaluated by western blotting with an anti-afadin pAb, and anti-GAPDH mAb as a loading control. (**B, C**) Cell counts and phase contrast images of endothelial cells. Confluent BMVECs and LMVECs were transfected with siRNA and scramble RNA, and then observed by light microscopy just after (0 day) and 2 days after transfection. Arrowheads in (**C**) indicate the elongated and shrunken cells with disrupted intercellular junctions. Bar graphs in (**C**) show the percentage of the elongated and shrunken cells among the total cells. Error bars in (**B**) and (**C**) indicate the mean ±S.D. from at least three independent experiments. N.S., not statistically significant. *, p<0.01 vs. BMVEC. (**D**) Increased F-actin staining in LMVECs by inactivation of afadin. At 2 days after transfection of siRNA and scramble RNA, BMVECs and LMVECs were immunostained with an anti-cleaved caspase-3 pAb. F-actin and nuclei were labeled with rhodamine-phalloidin and Hoechst 33258, respectively. Arrows indicate enhanced F-actin assembly, and arrowheads indicate cleaved caspase-3-positive cells. Bar graphs show the percentage of cleaved caspase-3-positive cells. Error bars indicate the mean ±S.D. from at least three independent experiments. Scale bars in (**C**) and (**D**) represent 100 µm and 50 µm, respectively.

We did not expect that targeted disruption of afadin in endothelial cells would cause severe defects of cell-cell junctions specifically in lymphatic vessels, but not in blood vessels, during mouse embryonic development. To address this issue, we conducted *in vitro* experiments using BMVECs and LMVECs, instead of primary endothelial cells derived from afadin cKO and control mice, because there is no established method to separate lymphatic endothelial cells from blood endothelial cells obtained from the mouse vessels. The expression of afadin was effectively knocked down at the concentration of 15 nM siRNA against afadin (**Fig. 4A**). Therefore, this concentration of siRNA was applied to further experiments. When confluent monolayers of BMVECs or LMVECs were transfected with afadin or scramble siRNA, a change of cell shape was observed only in LMVECs transfected with afadin siRNA for 2 days, although the cell count of each monolayer was not significantly changed in the experimental period (**Fig. 4 B and C**). The number of elongated and shrunken cells in the LMVEC monolayers was significantly increased compared with that in the BMVEC monolayers by knockdown of afadin, leading to disruption of intercellular adhesion as observed in afadin cKO lymphatic endothelial cells. This phenotype did not appear to be caused by apoptosis, because the degree of apoptosis evaluated by staining of cleaved caspase-3 was not significantly different between BMVECs and LMVECs (**Fig. 4D**). On the other hand, F-actin assembly was remarkably enhanced, especially at the cell periphery, in afadin-knockdown LMVECs, compared with that in afadin-knockdown BMVECs (**Fig. 4D** **, arrows**).

**Figure 5:**
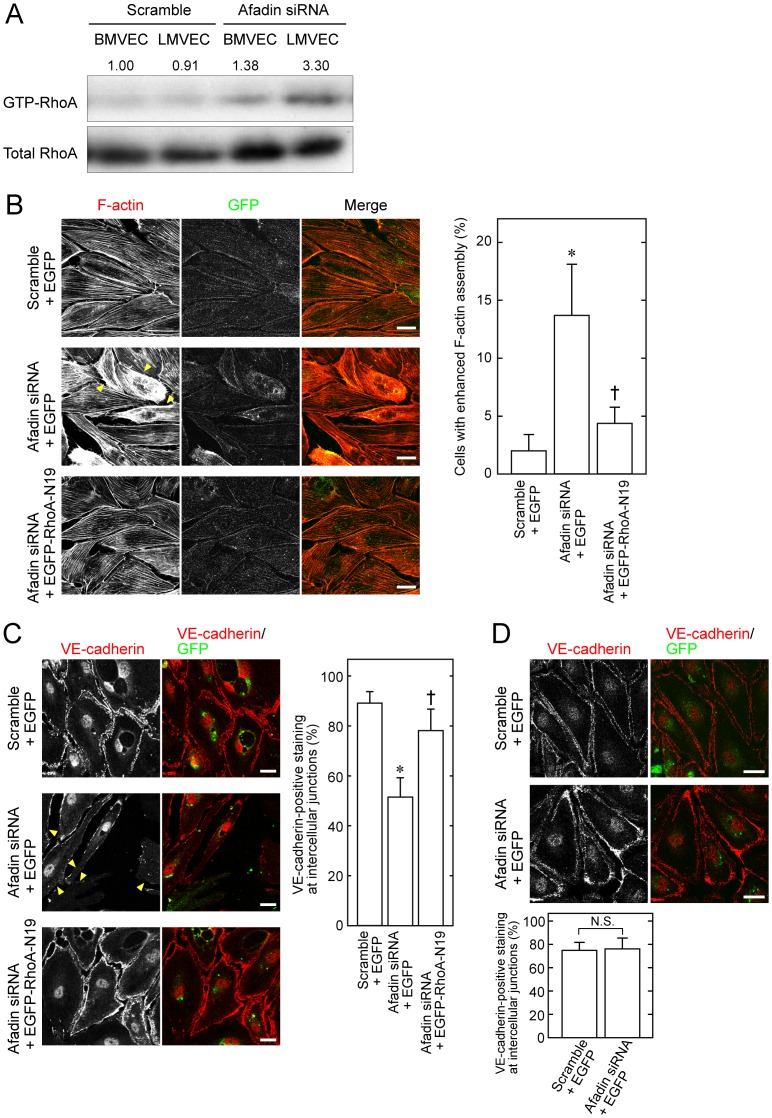
Enhanced activation of RhoA in LMVECs by inactivation of afadin. (**A**) Activation of RhoA in BMVECs and LMVECs. At 2 days after transfection of siRNA or scramble RNA, BMVECs and LMVECs were lysed and then used for the pull-down assay, followed by western blotting with an anti-RhoA pAb. The numbers above the blot represent the relative density of GTP-bound RhoA normalized to the total amount of RhoA by comparing the value of BMVECs transfected with scramble RNA, which is expressed as 1.00. (**B, C**) Restoration of cell morphology and VE-cadherin-mediated cell-cell junctions by introduction of dominant-negative RhoA in afadin-knockdown LMVECs. LMVECs transfected with the indicated combination of siRNA, scramble RNA, pEGFP and pEGFP-RhoA-N19 were labeled with rhodamine-phalloidin to visualize F-actin (**B**) and with an anti-VE-cadherin mAb (**C**). Arrowheads indicate enhanced F-actin assembly (**B**) and reduced VE-cadherin staining (**C**). (**D**) VE-cadherin-mediated cell-cell junctions in control and afadin-knockdown BMVECs. BMVECs transfected with scramble RNA + pEGFP or siRNA + pEGFP were immunostained with an anti-VE-cadherin mAb. Bar graphs show the percentage of cells with VE-cadherin staining at cell-cell junctions. Bar graphs in ***B***, ***C*** and ***D*** show the percentage of cells with enhanced F-actin assembly (**B**) and with VE-cadherin staining at cell-cell junctions (**C**) and (**D**). Error bars indicate the mean ±S.D. from at least three independent experiments. *, p<0.01 vs. Scramble +EGFP, and †, p<0.01 vs. Afadin siRNA +EGFP. N.S., not statistically significant. Scale bars represent 20 µm.

Activation of RhoA small G protein is reported to essentially regulate F-actin assembly [27], and excessive activation of RhoA can induce strong contractility that shrinks cells [21], [28], [29]. We performed a pull-down assay to examine RhoA activation in BMVECs and LMVECs. The amount of GTP-bound RhoA, which is the active state of RhoA, was increased by knockdown of afadin in both BMVECs and LMVECs, and more importantly, RhoA activation in afadin-knockdown LMVECs was apparently higher than that in BMVECs (**Fig. 5A**).

We finally examined whether the enhanced activation of RhoA is essentially associated with the phenotype observed with loss of afadin. When LMVECs were co-transfected with siRNA against afadin and pEGFP or pEGFP-RhoA-N19, a vector that expresses an EGFP-tagged dominant negative mutant of RhoA, the abnormal cell morphology observed by transfection of siRNA and pEGFP was almost recovered in cells transfected with siRNA and pEGFP-RhoA-N19 (**Fig. 5B**). Immunostaining with VE-cadherin also showed that the staining observed at the cell-cell junctions in control scramble siRNA-transfected LMVECs was markedly reduced in afadin-knockdown LMVECs, which was rescued by additional transfection of pEGFP-RhoA-N19 (**Fig. 5C**). In contrast, the VE-cadherin staining was not significantly affected in BMVECs even if afadin was knocked down in these cells (**Fig. 5D**). These results suggest that loss of afadin induces the activation of RhoA more strongly in lymphatic endothelial cells than that in blood endothelial cells, which may result in the occurrence of obviously abnormal phenotypes specifically in lymphatic vessels.

## Discussion

For the first time, we have shown that afadin in endothelial cells is essential for lymphatic vessel formation in the developing mouse embryo. This result is likely to be a new insight into lymphangiogenesis. During embryonic development, lymphatic vessels are formed by sprouting lymphatic endothelial cells from cardinal veins [30]. In mice, such sprouting begins at E10.5 [31], and lymphatic endothelial cells differentiate from vascular endothelial cells [26], [32], [33]. Although it has been reported that afadin is expressed in lymphatic endothelial cells as well as blood ones [17], [34], we did not expect that loss of afadin would more specifically affect the morphology of lymphatic vessels than that of blood vessels, because previous studies have demonstrated that mice deficient for other scaffolding molecules, such as β-catenin and p120^ctn^, at cell-cell adhesion sites in endothelial cells show embryonic lethality due to abnormal morphogenesis of blood vessels [35], [36]. Considering our results and those from other research groups, the scaffolding proteins at intercellular junctions appear to have respective functions for vasculogenesis and angiogenesis in blood and lymphatic vessels, although the localization of these proteins is very close and they cooperatively play roles in the formation of cell-cell junctions between endothelial cells [7].

In addition to afadin, several molecules critically affect lymphangiogenesis. For example, Aspp1, which is expressed specifically in endothelial cells and modulates the transcriptional activity of p53, preferentially regulates the formation of lymphatic vessels rather than blood vessels [20], [37]. It is also well known that vascular endothelial growth factor (VEGF)-C plays an essential role in the formation and function of lymphatic vessels via VEGF receptor-3 [38]. The study using VEGF-C-null mice showed that similar to our results, the null mice were severely edematous and embryonically lethal between E15.5 and E17.5 due to the failure of lymphatic vessel formation [39]. In the VEGF-C-null mice, the migration of lymphatic endothelial cells from cardinal veins, which is the initial step in lymphatic development, is inhibited. Although the steps on which VEGF-C and afadin function for the lymphatic development are different, it might be intriguing that both VEGF-C and endothelial-specific afadin deficient mice result in death at the similar embryonic period. Mice deficient for Syk, a non-receptor tyrosine kinase expressed in hematopoietic cells but not in mature endothelial cells, die of blood-lymphatic shunts [40]–[42]. The results obtained from Syk-null mice indicate that hematopoietic cells can control lymphangiogenesis by acting as endothelial progenitor cells. In addition to these previous studies, our data show that a scaffolding protein, afadin, is also implicated in the proper morphogenesis of lymphatic vessels.

Afadin plays a cooperative role with nectins, CAMs that bind to afadin, in the formation of cell-cell junctions. However, knockout mice for each of the nectin family members are viable and show no defects in both lymphatic and blood vessels [43]–[45]. Because the nectin family consists of four members, nectin-1 through nectin-4, knockout of one member may cause only small phenotypic changes. Thus, afadin, which is a partner of all the nectin family members, is likely to exert greater effects on lymphatic vessel development than those of each nectin. In addition to its involvement in the formation of intercellular junctions, afadin has diverse cellular functions including cell survival and regulates various signaling pathways [12]. Based on these data, we examined how afadin acts during the development of lymphatic vessels. Although lymphatic vessels are formed from veins as mentioned above, no significant difference was observed in the differentiation ability of lymphatic endothelial cells, which was evaluated by Prox1 staining, between control and afadin-knockout embryos. The proliferation ability of lymphatic endothelial cells, which was evaluated by BrdU incorporation, was also similar between the two types of embryos. In addition, the occurrence of apoptosis was unchanged in afadin-knockdown cultured lymphatic endothelial cells, compared with that in control cells. This result is inconsistent with that from a previous study [12]. Although a precise explanation for such a discrepancy can not be currently provided, the difference in the cell types may be associated with the vulnerability to the induction of apoptosis, in which endothelial cells may be more resistant than the fibroblasts used in the previous study.

In contrast to our previous study that demonstrated the involvement of afadin in angiogenesis in the retina using a small number of new born mice (P4–P7) [18], the most intriguing finding in this study is the phenotype induced by endothelial cell-specific ablation of afadin, which showed defects in lymphatic rather than blood vasculature in embryos. To address the different functions of afadin between lymphatic and blood endothelial cells, we focused on the activation levels of RhoA in these cells, because afadin has been reported to modulate RhoA activation [21]. We found that the activity for RhoA was remarkably higher in lymphatic endothelial cells than that in blood endothelial cells when afadin expression was ablated. Furthermore, the introduction of a dominant-negative RhoA into afadin-inactivated lymphatic endothelial cells restored the cell morphology, thereby providing the evidence that lymphatic endothelial cell-specific disturbance of intercellular junctions may be caused by increased RhoA activation induced by loss of afadin. However, the reason for different RhoA activation states between lymphatic and blood endothelial cells remains elusive. Afadin modulates RhoA activation via ARAP, one of the GTPase-activating proteins for RhoA [21]. The expression levels or functions of such GTPase-activating proteins that cooperate with afadin might be different between the endothelial cells of lymphatic and blood vessels. Further studies will be needed to resolve this issue.

The role of afadin in the process of the formation of cell-cell junctions is shown to be essentially mediated by the rearrangement of the actin cytoskeleton [4], [7]. Other research groups also reported that the actin cytoskeleton is important for the accumulation of VE-cadherin at the cell-cell contact site [46], [47]. Thus, the aberrant VE-cadherin staining pattern in lymphatic vessels observed in this study is considered to be via the abnormal actin cytoskeletal organization induced by the lymphatic endothelial cell-specific hyperactivation of RhoA in afadin cKO mice. On the other hand, if the aberrant VE-cadherin assembly is directly due to the depletion of afadin, such aberrant phenotype would be seen in both blood and lymphatic vessels, but actually it was seen only in the lymphatic vessels. Based on the previous and current results, it could be concluded that the aberrant VE-cadherin staining in lymphatic vessels of afadin cKO mice is mediated by the altered organization of the actin cytoskeleton.

As for the clinical implications, it is unclear that the deficiency of afadin is related to the adult-onset cardiovascular diseases, such as atherosclerosis and coronary heart diseases, because almost all of afadin cKO mice are embryonically lethal and thus it is difficult to conduct experiments using adult afadin cKO mice to elucidate the effects of afadin on such diseases. However, there is evidence that in our previous research [18], blood flow recovery in ischemic limbs was impaired in heterozygous afadin cKO mice compared with control mice, providing some therapeutic potential that improvement of the afadin function in the endothelium might be effective for patients suffering from ischemic diseases. Future translational and clinical studies are necessary to address this possibility.
